# Artificial Intelligence and Medical Education (2013–2024): A Scopus-Based Bibliometric Analysis

**DOI:** 10.24248/eahrj.v9i1.817

**Published:** 2025-09-30

**Authors:** Festus Mulakoli, Edward Misava

**Affiliations:** a School of Nursing and Midwifery, Aga Khan University, Kenya; b Network of Quality, Teaching and Learning, Aga Khan University, Kenya.

## Abstract

**Background:**

Artificial Intelligence (AI) is transforming medical education by enabling personalised learning, adaptive feedback, simulation-based training, and automated assessments. While AI offers significant benefits, including curriculum optimisation and virtual tutoring, concerns around data privacy, access, and ethical implementation persist. Although bibliometric studies have explored AI in healthcare, comprehensive analyses of global collaboration and publication trends in AI-focused medical education remain limited.

**Aim:**

This study aims to analyse global research trends, key contributors, and thematic developments in the application of AI within medical education.

**Methods:**

A bibliometric analysis was conducted using the Scopus database. The search strategy included terms such as “Medical Education”, “Artificial Intelligence”, “Machine Learning”, “Deep Learning”, “Clinical Training”, “Virtual Patients”, and “Simulation”. Data were analysed using the Bibliometrix R package to assess publication volume, keyword co-occurrence, author collaboration, and citation patterns.

**Results:**

Research output on AI in medical education has grown significantly, peaking in 2024 with 1,081 publications. The United States leads in publication volume, followed by Russia and Canada. “Artificial Intelligence” was the most frequently used keyword. Co-authorship and co-citation networks revealed strong international collaboration, with emerging themes in clinical competence, virtual simulation, and ethical considerations.

**Conclusion:**

The future of artificial intelligence in medical education is promising, with applications in personalised treatment plans, drug development, and virtual healthcare assistants. AI has transformative potential for medical education, particularly in personalised learning and simulation-based training. Strategic investment in AI literacy, ethical frameworks, and infrastructure is essential to ensure equitable and effective integration across global contexts.

## BACKGROUND

Artificial Intelligence (AI) has emerged as a transformative force in education, revolutionising teaching and learning methods at various levels. Its applications in medical education include intelligent tutoring systems, personalised learning platforms, automated grading, and adaptive learning systems.^1^ These tools have shown promising results in enhancing student learning outcomes, improving academic achievement, and increasing engagement in most medical training institutions worldwide.^2^

The integration of AI into medical education is particularly relevant in addressing persistent challenges such as limited access to expert instructors, variability in clinical exposure, and the need for scalable training solutions. AI technologies, including machine learning algorithms and natural language processing (NLP), have supported adaptive learning, automated assessments, and simulated complex clinical scenarios, thereby improving both the efficiency and quality of medical training.^3–10^

Several institutions of higher education have integrated AI-guided pedagogy into their teaching approaches. For instance, in the United States, most medical training institutions have platforms such as Body Interact and Osso VR, which use AI-driven virtual patients to simulate clinical scenarios in student practice. ^11^ In India, medical tertiary colleges use platforms such as MediSim, which uses AI for adaptive quizzes and case-based learning modules for NEET-PG preparation. ^12^ In the UK, Cambridge University uses an AI examiner to provide instant feedback on clinical cases, thereby reducing the grading workload of its faculty. Brazil's institutions of higher learning use the Sofia AI to assist in their OSCE assessment through NLP analysis. 13 However, all these institutions have reported challenges such as algorithmic bias, data privacy, and human oversight needs. ^14^ Integration of AI into medical education promises to enhance training efficiency and improve patient care amid all existing obstacles. ^15^

Similar trends have been observed in Africa, where medical training institutions continue to embrace artificial intelligence modalities in their teaching approaches. This adoption bridged gaps in training, improved access to quality learning, and enhanced the development of clinical skills. ^16^ Some important AI applications include virtual simulations, intelligent tutoring systems, and automated assessments that address the challenges associated with the workforce and infrastructure over time.^17^ For instance, the University of Cape Town (UCT) used AI-driven Touch Surgery to train medical students through interactive surgical simulations, which showed a 25% improvement in procedural accuracy.^18^ Kwame Nkrumah University (KNUST), another African institution, used the AutoMed Examiner to grade clinically relevant evaluations. ^19^ However, several gaps remain in the literature, particularly regarding the implementation of AI in low-resource settings, where technological infrastructure is often inadequate.^20^

Ethical concerns, such as data privacy^21^ and algorithmic bias ^22^, have also been underexplored in the context of medical education in most countries. Furthermore, few studies have evaluated the long-term impacts of AI tools on clinical competence and patient outcomes.^23,24^ Other challenges experienced by institutions within low-resource settings include inadequate Internet connectivity, prohibitive costs, and a lack of technical expertise. A 2023 study published in The Lancet Digital Health indicated that only 22% of medical schools in sub-Saharan Africa possessed reliable Internet access, significantly limiting the utilisation of cloud-based AI learning platforms.^25^ Furthermore, proprietary AI tools such as virtual anatomy tutors often require annual licensing fees exceeding $50,000, rendering them inaccessible to numerous institutions in low- and middle-income countries.^26^ To mitigate these challenges, researchers have advocated for scalable, cost-effective AI solutions such as the OpenAnatomy Project, which offers free, offline-compatible 3D anatomy models and has been successfully implemented in medical schools in Kenya and Nepal.^27^ Collaborations between academic institutions and technology firms have also proven to be effective. For instance, Makerere University in Uganda partnered with GE Healthcare to incorporate AI-powered ultrasound simulators into its curriculum, reducing training costs by 70%.^28^ These initiatives illustrate that, with strategic investments in digital infrastructure and localised AI tools, medical education in resource-constrained regions can overcome technological and financial barriers.

However, artificial intelligence is not limited to student-oriented applications. It also benefits educators by automating administrative tasks and providing real-time feedback. Moreover, artificial intelligence-powered tools such as virtual reality simulations and learning analytics dashboards are expanding the possibilities of educational technology.^29,30^ However, integrating artificial intelligence into education is challenging. Concerns have been raised regarding privacy issues, algorithmic biases, and the potential replacement of human educators.^31^

The digital divide and equitable access to artificial intelligence (AI)-powered educational tools remain significant challenges, particularly for the teaching fraternity in Africa.^32^ Many institutions face infrastructural limitations, such as unreliable internet connectivity, lack of access to modern devices, and insufficient technical support. These barriers hinder educators and students from fully leveraging AI-enhanced learning platforms.^33^ Moreover, disparities in digital literacy among faculty members further complicate the integration of AI into teaching practices. Addressing these challenges requires targeted investments in digital infrastructure, capacity-building programmes, and inclusive policies that prioritise equitable access to educational technology.^34^ Although artificial intelligence shows great promise in enhancing educational experiences and outcomes, its implementation must be approached with caution. A balanced strategy is essential, one in which AI complements rather than replaces human educators.^35^ Human teachers bring empathy, clinical judgement, and contextual understanding that AI cannot replicate. Overreliance on AI tools may risk depersonalising education and undermining the development of critical thinking and interpersonal skills.^36^ Therefore, AI should be integrated as a supportive tool to enhance teaching effectiveness, facilitate personalised learning, and reduce administrative burdens, while preserving the irreplaceable role of educators in shaping professional identity and ethical practice.^37,38^

Future research should focus on understanding the long-term effectiveness of artificial intelligence in education, addressing ethical concerns, and ensuring equitable access to artificially powered learning tools.^34^ Collaboration among technologists, educators, and policymakers is vital for shaping the ethical and equitable integration of artificial intelligence into global educational systems.^39–41^ Artificial intelligence (AI) has been poised to significantly impact medical education and healthcare delivery. There is a growing consensus among medical students and faculty that artificial intelligence should be incorporated into the medical school curriculum.^42,43^ However, current medical education has not kept pace with the rapid advancements in Artificial Intelligence, leaving future physicians potentially ill-equipped to manage artificial-intelligence-driven technologies and decision-making.^44,45^ While support for artificial intelligence in medical education is widespread, few medical students have received formal training on this subject.^42^ This gap exists despite students’ knowledge of artificial intelligence applications in clinical medicine and ethics. The lack of artificial intelligence integration in medical curricula is attributed to factors such as insufficient faculty expertise and a lack of guidance from accrediting bodies.^44^ To address these challenges, medical schools should incorporate artificial intelligence as a longitudinal tool in their curricula. This should include teaching artificial intelligence tools, algorithm development, data science, and the ethical implications of artificial intelligence in medicine.^46^ Innovative approaches, such as hackathon-style projects and multidisciplinary education involving computer science students, have been suggested.^42,47^ Additionally, faculty development programmes focused on artificial intelligence are necessary to bridge this knowledge gap.^48^ By integrating artificial intelligence education, medical schools have better prepared future physicians to leverage artificial strengths, manage their risks, and ensure a patient-centred digital future in medicine.^42,49^

Recent bibliometric analyses have revealed a growing body of literature on the application of AI in medical education.^50,51^ The United States, China, and Canada lead in publication volume, with institutions such as Harvard Medical School and the University of Toronto contributing significantly.^52,53^ The key themes include AI-enhanced diagnostics, virtual reality simulations, and intelligent tutoring systems.^54,55^ Journals such as the Journal of Medical Internet Research in Medical Education and Frontiers in Medicine have published influential studies on this topic, reflecting its increasing academic and practical relevance.^56,57^ Conducting a bibliometric study on artificial intelligence in medical education necessitates the application of statistical methodologies to analyse the developments, correlations, and impact of scholarly works on the integration of artificial intelligence in medical training. This process typically involves utilising academic databases such as Scopus or Web of Science to identify relevant publications and assess their references, contributors, subject terms, and publication patterns across various temporal periods.^58^ This study aimed to address these gaps by conducting a bibliometric analysis of global research on AI in medical education. By mapping publication trends, identifying leading contributors, and highlighting emerging themes, this study provides a comprehensive overview of this field. These findings will inform future research directions and support the development of equitable, effective, and ethically sound AI-driven educational strategies.

### Aim

To explore and analyse current publishing trends and research developments in the application of artificial intelligence in medical education and to identify key themes, contributors, and emerging directions in the field.

### Objective

To systematically analyse the global research landscape on the application of artificial intelligence in medical education by identifying its most common uses, leading contributors (countries and institutions), emerging trends, and future directions.

## METHODS

### Study Design

This study employed a bibliometric analysis methodology to examine publication trends related to artificial intelligence in medical education. Bibliometric data were retrieved from the Scopus database and analysed quantitatively using the Bibliometrix package in R. This software offers a comprehensive suite of tools for bibliometric analysis, including citation analysis, keyword co-occurrence mapping, and trend analysis of scholarly outputs. The results provide a structured overview of research activities, highlighting key themes, influential publications, and emerging areas within the intersection of artificial intelligence and medical education.

### Data Collection Inclusion and Exclusion Criteria

Articles were sourced from Scopus, a leading database renowned for its extensive coverage of peer-reviewed literature and its rich citation data. The inclusion criteria were limited to indexed publications published between 2015 and 2024. For an article to qualify, it had to contain at least one of the following keywords: “Artificial Intelligence”, “Machine Learning”, “Deep Learning”, “Medical Education”, “Clinical Training”, “Virtual Patients”, or “Simulation” within its title, abstract, or keyword list. Publications that were not accessible online were excluded from analysis.

### Database Selection

The selection of the Scopus database was guided by the focus of the study and the need for access to high-quality, peer-reviewed publications. Scopus was used to identify articles related to artificial intelligence in medical education. A carefully curated set of keywords was used to ensure comprehensive coverage of the relevant literature while minimising the inclusion of unrelated studies. This strategic approach helped capture the breadth of research in the field without introducing excessive noise.

### Search Strategy

A structured search strategy was developed for the Scopus database to identify the relevant literature on artificial intelligence in medical education. The search incorporated the terms “Medical Education” AND “Artificial Intelligence” OR “Machine Learning” OR “Clinical Training” OR “Virtual Patients” OR “Simulation”, aligned with the study's inclusion and exclusion criteria. Boolean operators such as AND, OR, and the asterisk (*) wildcard were employed to enhance the comprehensiveness of the search. Adjustments were made to the search syntax and indexing parameters to ensure consistency and accuracy across databases.

### Data Extraction and Visualization

For each article, key bibliographic information was extracted, including title, author names, author affiliations, abstract, publication date, and keywords. Two reviewers independently assessed the comprehensiveness and consistency of the extracted data to determine the relevance and suitability of each manuscript for inclusion. The duplicate records were identified and removed. Additionally, citations lacking the essential bibliometric metrics required for analysis were excluded to maintain the integrity and quality of the dataset.

### Statistical Analysis

A bibliometric analysis was conducted using Biblioshiny, an interactive web interface of the Bibliometrix R package, to generate and visualise networks of co-authorship, co-citation, and keyword co-occurrence. This approach facilitated the identification of research themes and clusters, which were subsequently labelled to represent major topics and subfields within the Scopus dataset. Data preprocessing, computation of bibliometric indicators, and generation of visual outputs were performed using the R Studio Software Version 4.4.2. This platform enables the calculation of collaboration indices, h-indices, and citation metrics, providing a comprehensive evaluation of research productivity and impact at the author, journal, and institutional levels. To create a detailed tag cloud, the Bibliometrix package was employed to extract and clean data from titles, abstracts, and keywords. Duplicate entries were removed, and text normalisation was performed to ensure consistency. The biblioanalysis function was used for an in-depth bibliometric evaluation, whereas the word cloud function visualised term frequency, with more frequently occurring terms displayed in larger font sizes.

International collaboration patterns were also analyzed using Bibliometrix based on author affiliation data from Scopus. Network visualization was generated to illustrate the co-authorship relationships between countries. In this map, line thickness represents the strength of collaborative ties, node size indicates publication volume, and distinct colors are assigned to different collaboration clusters using a bibliometric clustering algorithm. The resulting visualization effectively highlighted global research collaboration trends within the dataset.

### Quality Assessment Procedures

A systematic quality assessment process was conducted to ensure the reliability and relevance of the included studies. Each retrieved article was evaluated based on predefined criteria, including completeness of bibliographic metadata, relevance to the research topic, and availability of citation metrics. Two independent reviewers assessed the articles for consistency and comprehensiveness of the extracted data, including the title, abstract, keywords, author information, and publication details. Discrepancies between the reviewers were resolved through discussion and consensus. Duplicate records were identified using automated filtering tools and manually verified before removal. Articles lacking essential bibliometric indicators such as citation counts, author affiliations, or keyword data were excluded from the final dataset to maintain analytical integrity. The quality of the bibliometric data was further validated by cross-checking Scopus indexing standards and ensuring uniform formatting across all entries. This rigorous assessment process ensured that only relevant, high-quality publications were included in the analysis.

### Ethical approval

This article does not include any studies involving human or animal participants.

## RESULTS

### Annual Research Output on Artificial Intelligence and Medical Education

**FIGURE 1:**
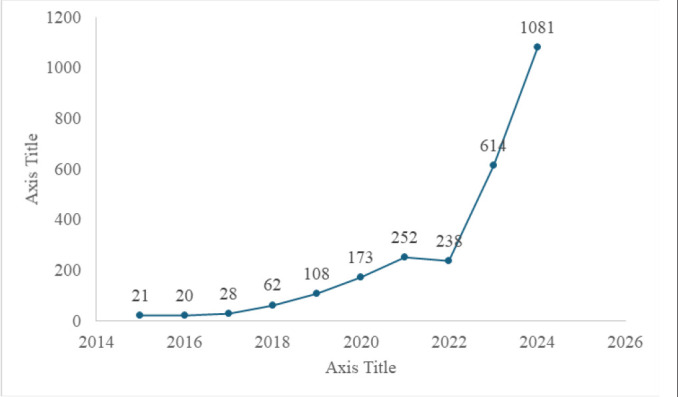
Number of Publications on Artificial Intelligence and Medical Education Per Year Between 2015–2024

The line graph shown in Figure 1 demonstrates an increasing trend in artificial intelligence and medical education publications from 2014 to 2024. This has consistently increased, indicating the ongoing interest and research in this area. Notably, there is a marked acceleration in growth after 2022 (238 to 1081 publications in 2024), reflecting a significant boost in research and development. The graph forecasts a peak in publications in 2024, with 1081 publications, hinting at the possible culmination of research efforts or a major milestone in the field. The overall trend was positive, suggesting a promising future for research and innovation at the intersection of artificial intelligence and medical education.

### Global Regions with Publications on Artificial Intelligence and Medical Education

**FIGURE 2:**
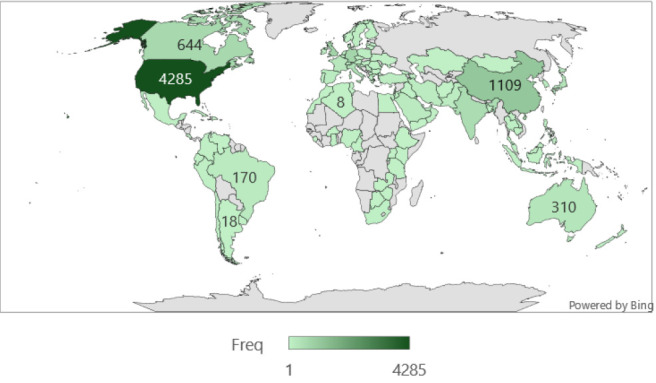
Geographic Trends in Publications on Artificial Intelligence in Medical Education, 2015–2024

As shown in Figure 2, the USA is the major contributor to research output on artificial intelligence and medical education, with 4285 publications. Other countries include Russia (n=1109), Canada (n=644), India (n=542), and Italy (n=470), which are the top five countries with the highest number of publications globally. South Africa (n=51) and Egypt (n=29) were the only African countries with the highest number of publications. Brazil (n=170) was the leading country in the South American region, with the highest number of publications.

### The Most Commonly Used Keyword in Publications

**FIGURE 3:**
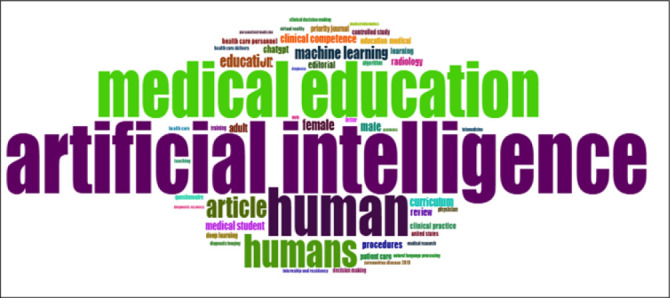
Schematic Visualization of the Most Frequently Used Keywords in Publications on Artificial Intelligence on Medical Education

Figure 3 illustrates the most frequently used keywords in the fields of artificial intelligence and medical education. Common keywords in cluster one were ‘artificial intelligence’, ‘education’, and ‘human’. This was followed by ‘Machine learning’ and ‘Articles’, which were the second-largest clusters. The third cluster contained keywords such as ‘clinical practice”, clinical competence”, “curriculum”, “radiology”, and “medical student”.

### Co-Occurrence Patterns Between Keywords

**FIGURE 4:**
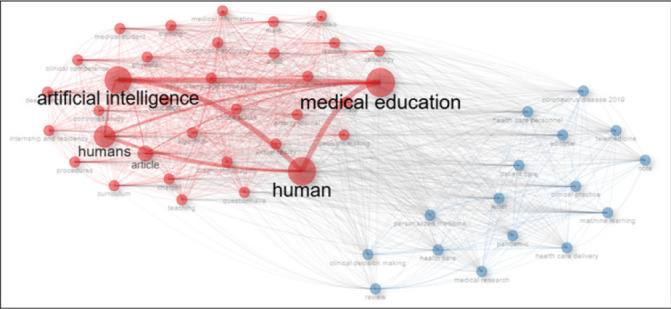
Co-Occurrence Network of Keywords in Publications on Artificial Intelligence in Medical Education

Figure 4 illustrates the co-occurrence patterns between the keywords related to artificial intelligence and medical education. Two main clusters were identified, with one cluster (red) dominated by three keywords: “artificial intelligence”, with a node connection to two other keywords, “medical education” and “human”. This is connected to other smaller clusters, which include keywords such as medical students, diagnostic imaging, physicians, procedures, teaching, internship/residency, training, medical informatics, and decision-making. The other cluster (blue) contained keywords such as coronavirus 2019, machine learning, personalised medicine, clinical practice, clinical decision-making, health care delivery, patient care, and reviews.

### Co-citation patterns in Artificial Intelligence and Medical Education

**FIGURE 5:**
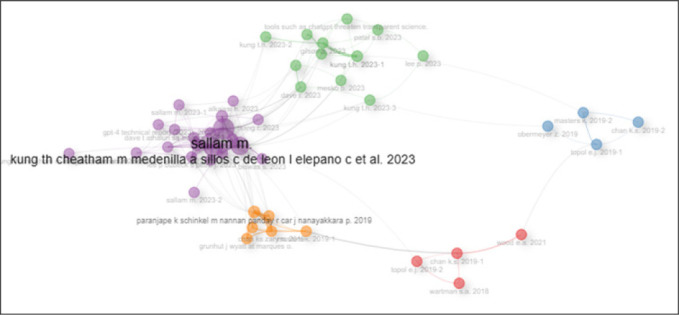
Co-Citation Network Illustrating Collaborative Relationships Among Authors in AI and Medical Education Research

Figure 5 presents a network visualisation of the co-citation patterns among different authors. Four clusters (Cluster 1-purple, Cluster 2-green, Cluster 3-orange, Cluster 4-blue, and Cluster 5-red in colour) can be identified. The node in cluster 1 identifies Sallam M as the main collaborating author. Another notable co-citation network is between Kung L. and Obermeyer Z., and then Lee P. and Master R., all from different clusters.

### Historiographic visualization of studies

**FIGURE 6:**
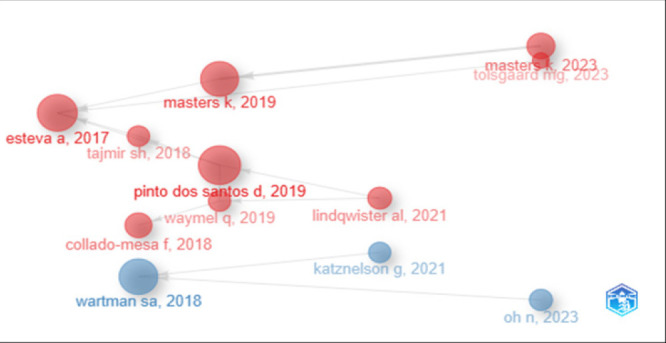
Historiographic Visualization of the Evolution of Research on Artificial Intelligence in Medical Education (2015–2024)

Figure 6 illustrates the evolution and citation dynamics of key AI research publications on artificial intelligence in medical education from 2017 to 2023. Each node in the timeline represents a published paper, with the size of the node indicating its impact or significance, likely based on the citation count or scholarly influence. The red and blue circles differentiate thematic or methodological clusters, possibly distinguishing between empirical studies (red) and conceptual or policy-oriented works (blue).

The earliest influential paper was Esteva et al. (2017), which appears as a large red node, suggesting foundational significance in the field. Subsequent impactful studies include Tajmir SH (2018), Collado-Mesa F (2018), and Wartman SA (2018)—the latter marked in blue, indicating a shift toward broader educational or ethical discussions.

The year 2019 shows a surge in publications, with notable contributions from Pinto Dos Santos D, Masters K, and Waymel Q, all in red, reflecting a growing empirical interest in integrating AI into medical training. Masters K reappears in 2023, indicating sustained scholarly engagement and influence.

Recent years (2021–2023) show diversification in research focus, with Lindqwister AL (2021) and Tolsgaard MG (2023) continuing the empirical trajectory, while Katznelson G (2021) and Oh N (2023) (both blue) suggest an expansion into ethical, pedagogical, or policy dimensions of AI in education.

### Collaborations Between Different Authors

**FIGURE 7:**
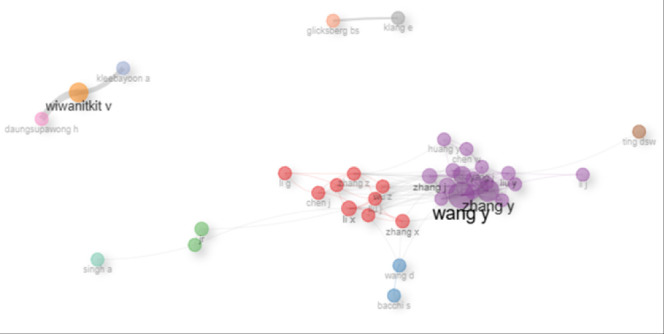
Schematic Presentation Author Collaboration Network in Publications on Artificial Intelligence in Medical Education

Figure 7 illustrates the collaborative landscape of the research community. The collaboration network graph visualises co-authorship patterns among researchers contributing to the field of artificial intelligence in medical education. Each node represents an individual author, whereas the edges between nodes indicate collaborative relationships through co-authored publications. The size of each name reflects the frequency or centrality of the collaboration, suggesting the relative influence or productivity of each researcher within the network.

The analysis revealed two main clusters that represent the main collaborating authors. The first largest cluster (purple) has Li X, Zhang X, and Wang D. Followed by the cluster (red) for Singh A, Li G, Zhang Z, and Chen J. The other clusters were Wiwantikit V, Daungsupawong H, and Kleebayoon A.

## DISCUSSION

The integration of artificial intelligence (AI) into medical education is reshaping the training, assessment, and support of future healthcare professionals. This bibliometric analysis revealed a sharp increase in AI-related publications between 2022 and 2024, consistent with the global trends observed in other studies.^59^ Countries such as the United States, China, and the United Kingdom, lead publication output, with institutions such as Harvard University, the University of London, and the National University of Singapore at the forefront of innovation.^60^ Comparative bibliometric studies have shown that while the United States maintains dominance in volume and citation impact, China has rapidly expanded its research output, particularly in areas such as large language models (LLMs), nursing education, and digital health.^61^ In India, surveys have revealed that although 74.8% of medical students supported structured AI training, only 3.7% felt confident in explaining AI to patients, highlighting the gap between awareness and competency.^59^ This mirrors the findings in Africa, where infrastructural limitations hinder AI adoption, despite growing interest and pilot implementation.

The historiographic visualisation in this study aligns with global patterns, showing a shift from foundational empirical studies to more diverse themes, including ethics, curriculum design, and virtual simulation. Similar bibliometric analyses have identified underexplored areas such as clinical reasoning and undergraduate education, suggesting opportunities for future research.^59,62^

The collaboration networks in this study revealed strong institutional ties, particularly between authors from China, India, and North America. These findings are consistent with the global bibliometric maps that show increasing cross-border partnerships, especially in AI-enhanced curriculum development and assessment tools.^63,64^

Keyword co-occurrence patterns in this study, dominated by terms like “artificial intelligence”, “machine learning”, and “clinical competence”, reflect a global thematic convergence. Other studies have noted an emerging interest in conversational agents (e.g., ChatGPT), medical ethics, and training methodologies.^65–67^ However, disparities remain in terms of curriculum integration. Some scoping reviews have emphasised the lack of standardised AI frameworks and the need for transversal skills, such as ethical awareness and digital competence.^66,68,69^

Africa's representation of AI-related medical education research remains limited, as shown in this bibliometric analysis. This under-representation reflects the persistent infrastructural and resource challenges across continents. However, it also highlights emerging centres of innovation that leverage AI to address local educational and healthcare needs. One such example is the University of Cape Town (UCT), which implemented Touch Surgery, an AI-powered surgical simulation platform.^70,71^ This innovation demonstrates the continent's capacity to develop scalable solutions that reduce faculty workload and enhance assessment reliability, although the formal citation data for this tool remain limited. This initiative has reduced training costs by 70%, making high-quality diagnostic training more accessible to students in low-resource settings. Makerere's AI Health Lab further supports this transformation by developing machine learning tools for point-of-care diagnostics, including pregnancy risk assessment and malaria screening. ^72^ While Africa's publication volume in AI medical education is modest, its contributions are strategic, impactful, and increasingly collaborative. With continued investment in infrastructure, faculty development, and international partnerships, African institutions are well-positioned to expand their role in shaping equitable and effective AI-driven medical education.

Despite this promising trajectory, several challenges persist. Ethical concerns, algorithmic bias, and unequal access to AI tools have echoed in global studies.^73^ For instance, while high-income countries invest in immersive technologies such as augmented reality (AR) or virtual reality (VR) for surgical training, low-resource settings struggle with the basic infrastructure. These disparities underscore the need for scalable, context-sensitive AI solutions and inclusive policy frameworks.^74^ Algorithmic bias in AI systems, often owing to under-represented data in training sets, has led to inequities in learning outcomes.^75^ Data privacy is another critical concern, particularly when AI tools handle sensitive patient information, and strict compliance with Health Insurance Portability and Accountability (HIPAA) and General Data Protection Regulation (GDPR) regulations is essential. Additionally, disparities in access to AI-enhanced education could widen the gap between well-resourced institutions and those with a limited technological infrastructure. Addressing these challenges will require collaboration among educators, AI developers, and policymakers to ensure responsible and equitable implementation.

Nevertheless, the integration of artificial intelligence (AI) into medical education has been poised to transform how future doctors and healthcare professionals learn and train. AI-powered adaptive learning platforms, such as Osmosis and Kaplan's MedSchool+, analyse individual student performance to deliver customised study materials and ensure optimal knowledge retention.^69,76,77^ These systems use spaced repetition algorithms, such as those in Anki, to reinforce complex medical concepts at scientifically determined intervals, thereby significantly improving the long-term recall.^78^ By tailoring content to each learner's strengths and weaknesses, AI is making medical education more efficient, engaging, and student-centred.

AI is also transforming assessments and tutoring in medical education. Natural language processing (NLP) tools such as ChatGPT and Google's Med-PaLM are used to simulate patient interactions, generate case-based questions, and provide instant feedback on clinical reasoning.^79^ Additionally, AI-driven platforms such as IBM Watson have automated grading for written and practical exams, reducing the faculty workload while maintaining assessment accuracy.^80^ Beyond tutoring, machine learning models analyse student performance data to predict learning outcomes, allowing educators to identify struggling students early and adjust their teaching strategies accordingly.^81^

The future of AI in medical education holds exciting possibilities.^82^ AI-powered virtual patients provide dynamic, interactive case scenarios that adapt to students’ decisions, offering a more realistic clinical experience. Federated learning and decentralised AI training approaches have helped improve algorithms without compromising data privacy. Establishing standardised regulatory frameworks is crucial for validating AI tools for medical training. As these innovations evolve, they promise to create a more personalised, immersive, and data-driven learning environment for the next generation of healthcare professionals. Beyond technological advancements, surging investments in AI healthcare solutions have played a pivotal role in driving research output. Major pharmaceutical companies and hospital networks are increasingly integrating AI into clinical workflows, from robotic surgery to AI-driven drug repurposing.^83,84^ Public-private partnerships and government initiatives, such as the NIH's AI in Healthcare grants, have further stimulated academic and industrial research. This influx of funding has not only expanded the volume of studies but has also encouraged interdisciplinary collaborations between computer scientists, clinicians, and policymakers.^85,86^

### Limitations and Challenges

The main limitation of this study is that it focuses on quantity metrics rather than the quality of published content. Some research works addressing crucial informational gaps in artificial intelligence in medical education may not have been featured in the top ten list created by the bibliometric analysis because of low citation counts. Second, only one database was searched. Several other databases have indexed articles on artificial intelligence and health informatics. Therefore, publications that were not indexed in Scopus may have been missing. Although the challenge of missing data could have been mitigated by performing separate analyses for each database, doing so was difficult. The Bibliometrix package in R Studio does not provide the full names of authors and the names of their affiliated institutions, which compelled us to manually search for the full names of the authors.

## CONCLUSION

In conclusion, AI reshapes medical education through personalised learning, simulation-based training, and automated assessments. However, its integration must be guided by ethical frameworks, inclusive policies, and strategic investments in infrastructure and capacity-building. Future research should prioritise longitudinal impact studies, cross-cultural evaluations, and the development of scalable, context-sensitive AI solutions to ensure that the benefits of AI in medical education are globally accessible and equitably distributed.
